# Modelling attenuation of irregular wave fields by artificial ice floes in the laboratory

**DOI:** 10.1098/rsta.2021.0255

**Published:** 2022-10-31

**Authors:** A. Toffoli, J. P. A. Pitt, A. Alberello, L. G. Bennetts

**Affiliations:** ^1^ Department of Infrastructure Engineering, The University of Melbourne, Parkville, Victoria 3010, Australia; ^2^ School of Mathematical Sciences, University of Adelaide, Adelaide, South Australia 5005, Australia; ^3^ School of Mathematics, University of East Anglia, Norwich NR4 7TJ, UK

**Keywords:** water waves, wave attenuation, wave–ice interaction

## Abstract

A summary is given on the utility of laboratory experiments for gaining understanding of wave attenuation in the marginal ice zone, as a complement to field observations, theory and numerical models. It is noted that most results to date are for regular incident waves, which, combined with the highly nonlinear wave–floe interaction phenomena observed and measured during experimental tests, implies that the attenuation of regular waves cannot necessarily be used to infer the attenuation of irregular waves. Two experiments are revisited in which irregular wave tests were conducted but not previously reported, one involving a single floe and the other a large number of floes, and the transmission coefficients for the irregular and regular wave tests are compared. The transmission spectra derived from the irregular wave tests agree with the regular wave data but are overpredicted by linear models due to nonlinear dissipative processes, regardless of floe configuration.

This article is part of the theme issue ‘Theory, modelling and observations of marginal ice zone dynamics: multidisciplinary perspectives and outlooks’.

## 1. Introduction

Ocean waves are a defining component of the marginal ice zone (MIZ) [1–5]. They regulate floe sizes and ice dynamics [6,7] in the MIZ, and hence ice extent and volume [8] (see also the reviews [9,10] in this issue). The ability to predict wave evolution in the MIZ, particularly wave attenuation over distance, is crucial to modelling wave-driven processes, such as ice break-up, and hence informing climate studies, evaluating ecosystem adaptation to climate change and planning the exploitation of natural resources in polar regions [11]. Field campaigns are critical to understanding and modelling fundamental physics, but the harsh MIZ environment makes *in situ* observations challenging [12–16]. As a complement or an alternative, laboratory experiments can be used to model complex ocean processes under controlled conditions, even in extreme sea states [17].

Laboratory experiments have a long tradition in the field of marine hydrodynamics, typically using model ice (saline or doped; see [18]). They have only relatively recently started to be used to investigate wave–floe interactions and wave propagation in the MIZ, to evaluate wave attenuation (and closely related wave transmission) [19,20] and concurrent wave forcing on floes, e.g. break-up [21,22] and rafting [23]. The majority of laboratory experiments on wave propagation in the MIZ employ artificial ice floes (e.g. plastic plates), as they are more versatile, e.g. do not require an ice tank and are easier to attach instruments to, while also providing a more compliant elastic response than does model ice, which exhibits unwanted plastic behaviour [24].

The laboratory experiments are often used to assess predictions given by theoretical models of wave–floe interactions and wave propagation in the MIZ. Use of artificial floes in the experiments is consistent with theoretical model assumptions in the so-called scattering regime, where floes are typically modelled as thin elastic plates when wavelengths ($L_{w}$) are shorter than floe lengths ($L_{f}$) and, hence, floes flex in response to wave forcing [25–29], or as rigid bodies when wavelengths are greater than floe lengths [30–35]. Scattering is negligible for wavelengths much greater than floe lengths ($L_{w}/L_{f}\gg 1$), and the floes are usually treated in theory as a continuum, resulting in a dispersion relation where, in contrast to energy-conserving scattering models, attenuation is created by a dissipative process [36–38], which makes the validity of artificial floes less clear. Almost all the theoretical models are linear, i.e. the results scale linearly with the incident wave amplitude on the basis of small steepness ($\varepsilon =\pi H/L_{w}$, where H is the wave height), and are treated in the frequency domain with regular incident waves.

For the sake of consistency with theoretical models and for simplicity, almost all laboratory experiments use regular incident waves. For instance, Bennetts *et al.* [39] analysed transmission of regular waves by a single square plastic floe in a wave basin. They found that the transmitted wave field becomes irregular for large incident steepnesses, and the transmission coefficient (i.e. the proportion of incident energy transmitted) tends to decrease with increasing steepness. They attributed the irregularity to the steeper incident waves (i) causing the floe to slam against the water surface and (ii) forcing water onto the upper floe surfaces because of their small freeboards, in a process known as overwash [29,40], thus creating high-frequency transmitted wave components. Moreover, they hypothesized that energy dissipation due to overwash and slamming reduces transmission, where the former was backed by a negative correlation between transmission coefficients and overwash depths.

Toffoli *et al.* [41] used measurements of reflection and transmission in wave flume experimental tests to show that incident wave energy is dissipated during interactions with the floe and that the proportion of dissipation increases with increasing steepness. Nelli *et al.* [42] extended the study to a wider range of tests and compared the results with tests where edge barriers were attached to the floe to prevent overwash, for which it was found that negligible wave energy is dissipated. Subsequent numerical modelling [43] and theory [44] give further support that overwash results in wave energy dissipation.

Bennetts and Williams [45] studied wave transmission through an array of circular wooden floes in a large wave basin. Tests were conducted for a low-concentration array involving 40 well-separated floes and a high-concentration array involving 80 densely packed floes. For the low-concentration array, linear attenuation models [46,47] were shown to predict transmission accurately for gentle incident waves (generally ε≤0.1) over $L_{w}/L_{f}=0.6-6.3$. Greater-steepness waves were tested at two wavelengths, and for the shorter wavelength, the larger steepness caused the transmission coefficient to decrease by 13%. The decrease was attributed to deeper and more energetic overwash, which was observed but not measured during the tests. For the high-concentration array, the linear model was shown to overpredict transmission, particularly for mid-range wavelengths, which was attributed to the strength and frequency of floe–floe collisions forced by the incident waves and backed by the analysis of accelerometer measurements on a subset of the floes. The finding has motivated subsequent experimental and numerical studies of wave-induced floe–floe collision properties [48] and their contribution to wave attenuation [49].

Ocean waves, such as those that propagate through the MIZ, are irregular and typically modelled as a superposition of regular components with random phases and different amplitudes, wavelengths and directions [17]. For linear systems, the regular wave components can be superposed to form the response for irregular wave forcing. However, the regular wave experiments discussed earlier indicate that the nonlinear processes of overwash, collisions and slamming occur in wave–floe interactions, even for relatively small-incident-steepness waves for which linear theory would usually be considered valid. Therefore, in this article, we revisit the experimental campaigns of [39,45] and analyse previously unreported tests involving irregular incident waves. We compare transmission for the regular and irregular tests, with a particular focus on the influence of nonlinear wave–ice interaction processes.

## 2. Single-floe experiments

### (a) Experimental set-up

**Figure 1.**
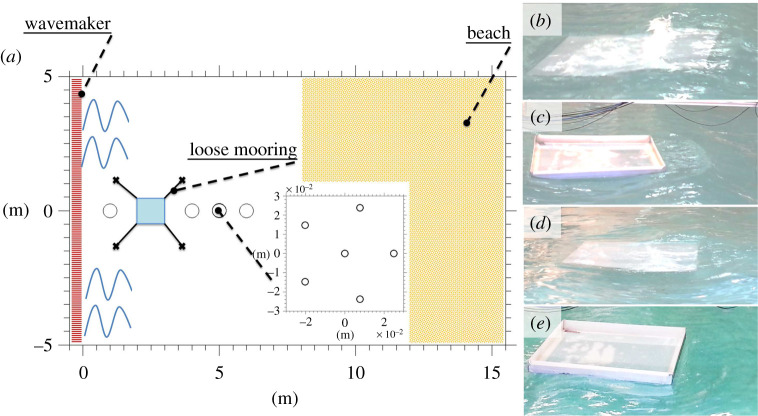
Experimental set-up (*a*) and snapshots of experiments involving irregular incident waves with $\varepsilon_{p}=0.15$: unidirectional tests (N=100) without (*b*) and with (*c*) edge barrier; directional tests (N=10) without (*d*) and with (*e*) edge barrier. (Online version in colour.)

An experiment was conducted in the wave basin at the Coastal Ocean and Sediment Transport laboratory at the University of Plymouth, UK. The basin is 10 m wide and 15.5 m long and was filled with fresh water 0.5 m deep (figure 1*a*). At one end of the basin, a wavemaker with 20 individually controlled active pistons generated incident waves. The pistons automatically adjust their velocities to absorb waves reflected by side walls or the floe. At the other end, a sandy beach with a 1 : 10 linear slope dissipated about 95% of the incoming wave energy (standard for linear profiles, e.g. [17,21]). Residual energy returning from the beach is absorbed by the active pistons, preventing the formation of persistent oscillations in the basin.

A square polypropylene floe with side lengths $L_{f}=1\;m$ and thickness 5 mm was deployed 2 m from the wavemaker (figure 1*a*). Its density of $905\;\mathrm{kg}\;m^{-3}$ and Young’s modulus of 1.6 GPa are comparable to those of sea ice, which has density varying from $720\;\text{to}\;940\;\mathrm{kg}\;m^{-3}$ and Young’s modulus ranging from 1 to 6 GPa [50] (no scaling factor is applied). The high density allows a small-freeboard, facilitating overwash (figure 1*b*,*d*) even in mild wave conditions [39]. A loose mooring was applied to avoid free drift. Tests were also conducted with edge barriers around the floe (as in [27,31,42]; see figure 1*c*,*e*).

Irregular waves were generated by imposing a JONSWAP spectrum at the wavemaker to model the spectral energy density in the frequency domain and a $\cos^{N}(\vartheta )$ directional distribution, where N is the directional spreading coefficient and ϑ is the wave direction. Random phases uniformly distributed within the interval [0,π) and random amplitudes distributed according to a Rayleigh distribution were used when converting spectral energy into piston displacements. The JONSWAP spectrum had peak wave period $T_{p}=0.8\;s$ and peak enhancement factor γ=3. Two incident wave field configurations of different strengths were tested, with significant wave heights $H_{s}=4\sqrt{m_{0}}=0.032\;m$ and 0.048 m (where $m_{0}$ is the spectral variance). These configurations define a peak wave steepness (a parameter controlling wave dynamics, wave breaking and wave-induced loads [17,51–53]) corresponding to $\varepsilon_{p}=\pi H_{s}/L_{w,p}=0.10$ and 0.15, respectively, where $L_{w,p}\approx L_{f}$ is the wavelength associated with the peak period through the linear dispersion relation [54]. The directional spreading coefficient was set to N=100 to model a unidirectional wave field and N=10 for a realistic directional sea state [17], noting that N switches naturally to a wavelength-dependent function after generation [53]. Regular wave fields with wave periods 0.6 s, 0.8 s and 1 s and different steepnesses were also tested, and results were reported by Bennetts *et al.* [39], although not for cases using edge barriers.

Capacitance gauges monitored the water surface elevation at 128 Hz sampling frequency and approximately 0.1 mm accuracy. One gauge was deployed 1 m in front of the floe to capture the incident and reflected waves. In the lee of the floe, three gauges were deployed every metre to track the transmitted wave field. At 2 m from the rear edge of the floe, a six-gauge array, arranged as a pentagon of radius of 0.25 m (figure 1*a*), was deployed to measure directional properties. Each incident wave field was tested for all artificial floe configurations (i.e. without and with edge barriers). In addition, benchmark tests were conducted for the incident wave fields in the absence of a floe. For irregular waves, 40 min time series were recorded to ensure enough data for statistically stable estimates of the wave spectrum, noting that spectral density functions measured in the basin compare well with the input counterpart (see electronic supplementary material, figure S1), despite some differences in the upper tail. For regular waves, only 5 min time series were measured owing to their deterministic nature.

### (b) Wave transmission in the wavelength-direction domain

**Figure 2.**
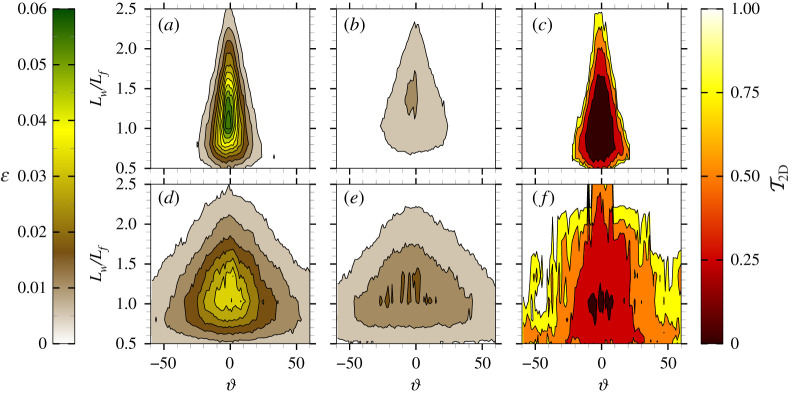
Plots of incident ε (*a*,*d*), transmitted ε (*b*,*e*) and $T_{\text{2D}}$ (*c*,*f*) as functions of normalized wavelength and wave direction for unidirectional (N=100) (*a*–*c*) and directional (N=10) (*d*–*f*) experiments with $\varepsilon_{p}=0.15$. (Online version in colour.)

Figure 2 shows the spectral densities of the steepness ε (*a*,*b*,*d*,*e*) and the transmission coefficient $T_{\text{2D}}(L_{w},\vartheta )=a_{\text{out}}(L_{w},\vartheta )/a_{\text{in}}(L_{w},\vartheta )$, i.e. the ratio of transmitted mode amplitudes ($a_{\text{out}}$) to the incident counterparts ($a_{\text{in}}$), with the amplitude being estimated from the spectral energy $E(L_{w},\vartheta )$ as $a(L_{w},\vartheta )=\sqrt{2E(L_{w},\vartheta )\Delta L_{w}\Delta \vartheta }$ (*c*,*f*), for the steepest irregular incident wave field ($\varepsilon_{p}=0.15$) and for unidirectional (N=100; *a*,*b*,*c*) and directional (N=10; *d*,*e*,*f*) cases. For the steepness, incident (*a*,*d*) and transmitted (for a floe without barriers; *b*,*e*) spectra are shown. The directional spectral density function is reconstructed with a wavelet directional method [55], using time series from the six-gauge array. The process is applied to windows of 256 data points with 50% overlap and an ensemble average is computed.

For the unidirectional incident wave field (figure 2*a*), energy is concentrated in a narrow directional band ($-10^{\circ }<\vartheta <10^{\circ }$), where the spectral components are steep and experience considerable energy loss during floe interactions [41], with less than 20% of their energy being transmitted (figure 2*c*). The dissipation results in substantial flattening of the spectral shape (figure 2*b*).

For the directional incident wave field (figure 2*d*), the energy is spread across a wide range of directions ($-60^{\circ }<\vartheta <60^{\circ }$). The spectral peak is less sharp, and modes carry less steepness than in the unidirectional case (approx. 40% less at the peak), making wave–floe interactions less vigorous. Major energy loss occurs for modes with high steepness (ε>0.035) over $0.8<L_{w}/L_{f}<1.4$ and $-10^{\circ }<\vartheta <10^{\circ }$ (figure 2*f*). Transmission increases with angle of propagation for $|\vartheta |>10^{\circ }$, as mode steepness decays. The non-uniform transmission rate smooths the spectral peak (figure 2*e*) without altering the spectral density at oblique directions, resulting in an increase of directional spreading.

Incident wave components that do not interact directly with the floe may be present in the transmitted field due to diffraction (in both unidirectional and directional sea states) or reflection from the side walls (in directional sea states), producing directional focusing and, hence, increasing the wave amplitude. Indeed, a weak increasing trend of wave energy is reported with distance from the floe (see electronic supplementary material, figure S2) for both undirectional and directional sea states. Nevertheless, the gain reported at the position of the six-gauge array is less than 3% relative to the measurements at the closest gauge and hence can be considered negligible.

### (c) Transmission coefficient

**Figure 3.**
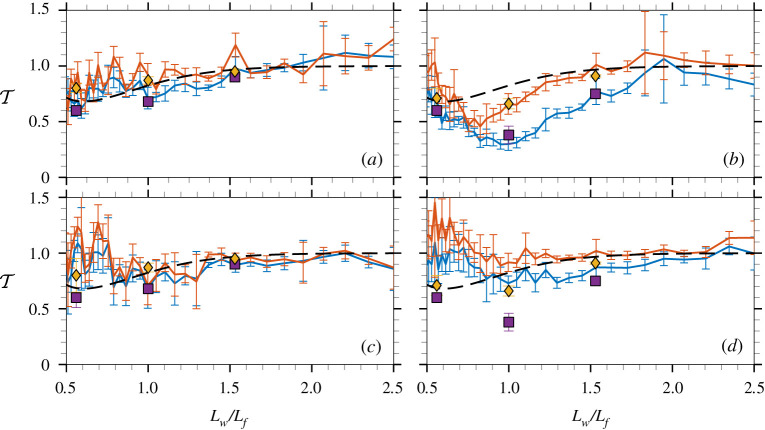
Mean and first standard deviation (error bars) of T as a function of normalized wavelength from experimental results for regular waves with (yellow diamonds) and without (purple squares) barriers and irregular waves with (orange line) and without (blue line) barriers compared with linear theory (dashed line). The experiments have N=100 and $\varepsilon_{p}=0.1$ (*a*), N=100 and $\varepsilon_{p}=0.15$ (*b*), N=10 and $\varepsilon_{p}=0.1$ (*c*), and N=10 and $\varepsilon_{p}=0.15$ (*d*), and regular waves have matching steepness. (Online version in colour.)

Figure 3 shows the transmission coefficient T, i.e. the average, direction-integrated version of $T_{\text{2D}}$, as a function of normalized wavelength, and includes error bars equivalent to one standard deviation from the ensembles to benchmark uncertainties. Results are shown for all unidirectional (N=100; *a*,*b*) and directional (N=10; *c*,*d*) incident wave fields, and the least energetic ($\varepsilon_{p}=0.10$; *a*,*c*) and most energetic ($\varepsilon_{p}=0.15$; *b*,*d*) cases. Results are also included for regular wave tests with steepness consistent with the steepness at the spectral peak and, thus, differ between the left- and right-hand panels, along with two-dimensional linear model predictions [56], which are identical for all panels.

For the unidirectional and least energetic incident wave field (N=100 and $\varepsilon_{p}=0.1$; figure 3*a*), the transmission coefficient increases almost monotonically with wavelength, in quantitative agreement with regular wave tests, and full transmission is reached for approximately $L_{w}/L_{f}>1.8$. Overwash was observed during the tests, but its effect on transmission is minor, as substantiated by the generally good agreement with linear theory and tests with barriers, for which transmission is less than 10% greater than in tests without barriers.

Broadening the directional spreading (N=10; figure 3*c*) does not have a notable effect for $L_{w}/L_{f}>0.8$, as indicated by the agreement with regular wave tests and linear theory. For $L_{w}/L_{f}<0.8$, the transmission coefficient is approximately 25% greater than in the unidirectional case, which is attributed to the floe slamming on the water surface, generating high-frequency wave modes (visible in figures 1*d*,*e* for $\varepsilon_{p}=0.15$), as noted previously in [39,42]. The transmission coefficient for the floe with barriers is less than 5% greater than the floe without barrier.

Wave floe interactions are most intense for the unidirectional and most energetic incident wave field (N=100 and $\varepsilon_{p}=0.15$; figure 3*b*). The interactions are characterized by intense overwash (figure 1*b*), which induces significant breaking dissipation over the floe [43]. The transmission coefficient decreases in comparison to the $\varepsilon_{p}=0.10$ case over most of the wavelength range ($L_{w}/L_{f}>0.7$). The decrease is greatest for $0.7<L_{w}/L_{f}<1.3$, which contains the steepest modes of the incident field, with less than 50% of the incident energy being transmitted. The decrease is much smaller for the floe with an edge barrier and negligible for $L_{w}/L_{f}>1.5$. The decrease, which is not visible in the regular wave data, is attributed to localized wave-breaking dissipation at the trailing floe end (figure 1*c*). The transmission coefficients for the irregular wave test agree with the corresponding transmission coefficients for the regular wave tests.

For the directional incident wave field (N=10; figure 1*d*), the decrease in transmission is much weaker, as the overwash is less intense. There is a pronounced increase in transmission for short wavelengths ($L_{w}/L_{f}<0.9$). It is similar to the increase in transmission in the less energetic case (*c*) and the unidirectional case (*b*), where the presence of overwash suppresses the increase.

## 3. Multiple-floe experiment

### (a) Experimental set-up

An experiment that investigated the transmission of unidirectional waves through an array of wooden floes was conducted in the Basin de Génie Océanique FIRST wave basin facility, located at Océanide, La Seyne sur Mer, France, for which Bennetts and Williams [45] reported the regular wave test results. The basin is 16 m wide and 40 m long and filled with fresh water 3.1 m deep. Waves are generated by the wavemaker at the left end of the basin, which propagate through the array of floes and are absorbed by a static beach of length 8 m at the far end. The water surface elevation to the left and right of the floe array was measured by 10 probes with a 25 Hz sampling frequency.

**Figure 4.**
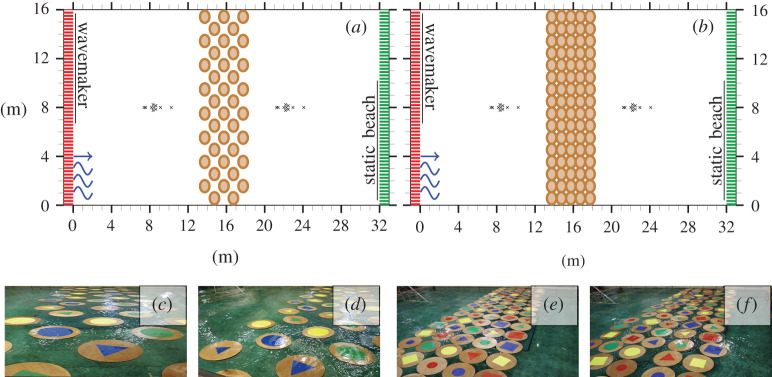
Experimental set-up for basin configurations with floe arrays of low (*a*) and high (*b*) concentrations. Photos taken during low-concentration (*c*,*d*) and high-concentration (*e*,*f*) array tests. (Online version in colour.)

Two arrays of wooden floes were studied: one with a low concentration of 38% (figure 4*a*,*c*,*d*) and the other with a high concentration of 77% (figure 4*b*,*e*,*f*). Both arrays were composed of circular floes with diameter 0.99 m and thickness 0.033 m. The Young’s modulus of the floes was 4 GPa, and the density of the floes was $545\;\mathrm{kg}\;m^{-3}$, which is lower than that of sea ice and, hence, gives the wooden floes a larger freeboard than ice (and polypropylene) floes. The floes were loosely kept in place using a mooring system that connected the centre of each floe to the bottom of the basin. The mooring had a natural period of 12.5 s, which was significantly longer than the studied wave periods. The mooring thus allowed the floes to respond with the natural motions predicted by a linear theory of wave action while maintaining the array between tests.

Regular and irregular wave tests were conducted for both arrays. The steepness of the regular incident waves was ε=0.02−0.13, with periods in the range of 0.65−2 s for tests that ran for 70 s. The irregular incident waves followed the JONSWAP spectrum with peak enhancement factor γ=3.3, peak steepness values $\varepsilon_{p}=0.02-0.08$ and peak periods of 0.8 s, 1.4 s and 2 s for tests that ran for 6.5 min (comparison of measured wave spectra and target wave spectra is provided in the electronic supplementary material, figure S1). All incident regular wave periods and irregular wave peak periods had corresponding calibration tests where no wooden floes were present.

### (b) Low-concentration array

**Figure 5.**
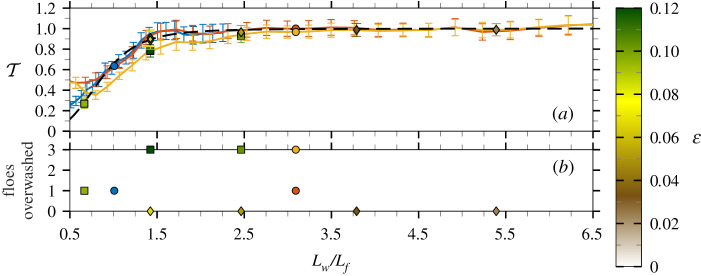
Mean and standard deviation (error bars) of T as a function of normalized wavelength for low-concentration experiments (*a*) for regular waves where overwash was (black squares) and was not (black diamonds) observed (coloured by ε) and for irregular waves with $\varepsilon_{p}=0.06$ (blue line), $\varepsilon_{p}=0.04$ (orange line) and $\varepsilon_{p}=0.08$ (yellow line) with $L_{w,p}$ shown (black circle). Observations of the number of floes overwashed during experiments (*b*), where irregular waves are plotted using $L_{w,p}$. (Online version in colour.)

Figure 5*a* shows the transmission coefficient at each mode, i.e. $T(L_{w})=a_{\text{out}}(L_{w})/a_{\text{in}}(L_{w})$, for all tests (regular and irregular) using the low-concentration array. The wave amplitude for each mode ($a(L_{w})$) was estimated from the spectral energy $E(L_{w})$ using $a(L_{w})=\sqrt{2E(L_{w})\Delta L_{w}}$. The spectral energy $E(L_{w})$ was measured from the time series of each transmitted probe by Fourier analysis and averaged over the probes to produce the mean transmitted amplitude for the test ($a_{\text{out}}$) and the corresponding incoming amplitude ($a_{\text{in}}$) from the calibration test, with the error bars denoting a standard deviation between probes [45].

Predictions from a two-dimensional linear attenuation model [47] are included as benchmarks. Figure 5*b* provides experimental observations of the number of floes overwashed in the direction of wave propagation for each test. Examples of the observed number of floes overwashed can be found in the electronic supplementary material, figure S3. In the low-concentration array, the maximum number of floes in the direction of wave propagation is 3, so three floes overwashed means all floes in the array were overwashed during the test. In the low-concentration array, no floe–floe collisions were observed, and so wave attenuation is attributed to scattering and dissipation in the floe–wave interaction.

The transmission coefficients for the irregular wave tests with $\varepsilon_{p}=0.04$ and 0.06 agree well for $L_{w}/L_{f}>0.8$, and also with the low-steepness regular wave tests (ε<0.1), where overwash occurred at most one floe deep into the array (figure 4*c*), and the linear model predictions. The agreement indicates that wave transmission through the array is a linear scattering process and not significantly affected by dissipation due to overwash. For shorter waves ($L_{w}/L_{f}<0.8$), the agreement between the low-steepness irregular wave tests ceases to hold, and both tests have larger T-values than predicted by the linear model, which indicates some transfer of wave energy to shorter wavelengths as observed for the single-floe tests in §2. The transfer is most noticeable in the $\varepsilon_{p}=0.04$ test, which is likely because the short wavelengths are further from the incident peak wavelength and, thus, had relatively low incident energy.

The irregular wave fields for the $\varepsilon_{p}=0.08$ and $\varepsilon_{p}=0.04$ tests have the same peak period. The larger steepness results in overwash of all floes in the array and a reduction in T of up to 16% due to dissipation. The transmission coefficient agrees with that of the high-steepness regular wave tests (ε>0.1, where overwash was observed for all floes in the array; figure 4*d*), which is likely because the regular waves have steepness comparable to the steepness of the corresponding components of the irregular field, but this agreement is not expected to hold in general, as overwash and other dissipative processes are nonlinear [43,44]. The transmission coefficient reduction is most significant for $0.75<L_{w}/L_{f}<3.5$. For $L_{w}/L_{f}>3.5$ the reduction is negligible, and for $L_{w}/L_{f}<0.75$ there is evidence of energy transfer, as for the lower-steepness tests.

### (c) High-concentration array

**Figure 6.**
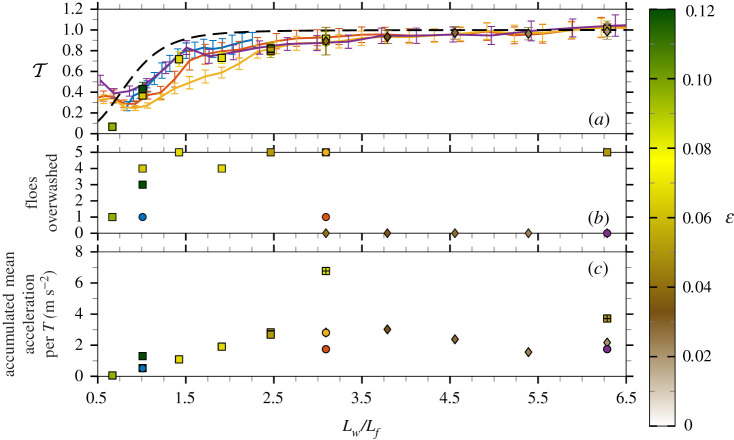
Plots in figure 5 repeated for high-concentration experiments with additional irregular wave experiment with $\varepsilon_{p}=0.02$ (purple line). Additional panel showing accumulated mean acceleration per wave period (peak period for irregular waves) from collisions (*c*) with experiments where the acceleration of one or multiple collisions saturated the signal (+). (Online version in colour.)

Figure 6*a*,*b* is identical to figure 5*a*,*b* but for the high-concentration array; examples of the observed number of floes overwashed are given in the electronic supplementary material, figure S4. Figure 6*c* demonstrates the accumulated mean acceleration due to floe–floe collisions per period measured by accelerometers fixed to floes in the middle column of the array. The accumulated mean acceleration combines the measures of frequency (collisions per period) and strength (mean acceleration for each collision) of collisions, which collectively summarize the collision behaviour [45].

Figure 6*a* shows that transmission for the high-concentration array tests is below the linear model predictions, except for $L_{w}/L_{f}<0.75$. For most wavelengths, the measured transmission values are bounded above by the irregular wave experiment with $\varepsilon_{p}=0.06$ and below by $\varepsilon_{p}=0.08$. From the overwash observations and collision measurements (figure 6*b*,*c*), the bounding experiments correspond to weak collisions and minimal overwash ($\varepsilon_{p}=0.06$; figure 4*e*) and strong collisions and total overwash ($\varepsilon_{p}=0.08$; figure 4*f*). Therefore, the reduction in transmission is correlated with the strength of overwash and collisions [42,48], noting that dissipation characteristics differ from those in the low-concentration tests, where no collisions occurred. Further, floe–floe collisions drive overwash of floes, as demonstrated by the incoming waves for which overwash was observed in the high-concentration array but not in the low-concentration array. The effect of dissipation in the experiments is most evident for $0.75<L_{w}/L_{f}<5.5$, i.e. collision-driven dissipation affects longer wave components. Energy transfer is again inferred for $L_{w}/L_{f}<0.75$. The addition of collision-driven dissipation alters the shape of the transmission curves, which can be seen by comparing the irregular wave tests with the same peak period and different steepnesses ($\varepsilon_{p}=0.04$ and $\varepsilon_{p}=0.08$), and contrasts with the effect without collisions (figure 5*a*).

## 4. Discussion and conclusion

Two independent experimental studies in three-dimensional wave basins have been revisited, for which irregular wave tests were conducted but not previously reported. One experiment tested irregular waves, including directional spreading, with two peak steepness values comparable to storm and polar cyclone conditions, interacting with a single square polypropylene floe with length close to the dominant wavelength (scattering regime). For storm conditions, wave–floe interactions were relatively weak, allowing more than 80% of energy to be transmitted for both unidirectional and directional waves. Overwash was observed but was not found to affect wave transmission, which is consistent with linear model predictions and regular wave tests. For cyclone-like conditions, wave–floe interactions and overwash were vigorous, forcing intense breaking dissipation to occur on the floe, especially in unidirectional waves, where less than 50% of the incident energy is transmitted. The unidirectional irregular wave and regular wave tests agreed well, and both were overpredicted by the linear model, which does not capture overwash dissipation. Directional spreading reduced the steepness of individual wave components, decreasing dissipation due to overwash and, hence, enhancing transmission.

The second experiment used arrays of wooden floes. Transmission of unidirectional irregular waves through low- and high-concentration floe arrays was tested at a range of peak periods and peak wave steepnesses spanning gentle to storm-like conditions. For the low-concentration array, transmission of gentle regular and irregular incident waves was predicted accurately by a linear model. Transmission decreased for storm-like waves because of dissipation effects correlated with the number of floes overwashed. For the high-concentration array, agreement was found between irregular and regular wave tests, but transmission was below model predictions, even for gentle steepness, which correlated with the strength of floe–floe collisions and overwash. The high-concentration array demonstrated collision-driven overwash, in contrast to current overwash models where overwash is due solely to individual floe interactions with large (steep) waves [40,44,57].

The experiments have key differences beyond the number of floes involved in the tests, such as the material densities used for the floes, which resulted in (unrealistically) large freeboard in the multiple-floe tests and, hence, different overwash onset thresholds. However, both experiments identify that (i) overwash, whether forced by wave impact on a single floe or collisions in an array of floes, is a driver of wave attenuation and (ii) across the full wave spectrum, overwash-driven dissipation is the most effective for wavelengths comparable with floe length. The shared findings from two independent experiments are evidence of their robustness. Further, there is good agreement between irregular and regular wave tests, especially when undirectional propagation is assumed. Directional spreading, nonetheless, creates inconsistencies with regular tests due to a reduced overwash. Knowledge of the directional wave spectrum is therefore important for achieving a more accurate estimation of wave–ice interaction processes in realistic oceanic conditions.

Contemporary global wave models compute wave attenuation in the MIZ with dissipation terms based on friction or viscoelastic processes [58,59] and linear scattering [60,61], but exclude collisions and overwash, in part because of a lack of direct *in situ* observations. Considering that floe sizes in the MIZ (10–100 m) are comparable to wavelengths of dominant (i.e. most energetic) wave components (approx. 25−350 m [62,63]), it is perhaps unsurprising that model predictions are reported to overestimate wave heights in the Arctic MIZ [58]. With large waves reported in high-concentration MIZs more than 50 km from the edge [14,15,20], an accurate estimation of wave attenuation in the MIZ is important to the evaluation of sea-ice dynamics. The challenge is to design new field studies and develop detailed models to resolve these mechanisms, to allow a generalization of the attenuation rate.

## Data Availability

The data can be downloaded at https://doi.org/10.5281/zenodo.6332923 [64]. Electronic supplementary material is available online [65].
